# Effects of conformational ordering on protein/polyelectrolyte electrostatic complexation: ionic binding and chain stiffening

**DOI:** 10.1038/srep23739

**Published:** 2016-03-31

**Authors:** Yiping Cao, Yapeng Fang, Katsuyoshi Nishinari, Glyn O. Phillips

**Affiliations:** 1 Glyn O. Phillips Hydrocolloid Research Centre, School of Food and Pharmaceutical Engineering, Faculty of Light Industry, Hubei University of Technology, Wuhan 430068, China; 2 Hubei Collaborative Innovation Centre for Industrial Fermentation, Hubei University of Technology, Wuhan 430068, China

## Abstract

Coupling of electrostatic complexation with conformational transition is rather general in protein/polyelectrolyte interaction and has important implications in many biological processes and practical applications. This work studied the electrostatic complexation between *κ*-carrageenan (*κ*-car) and type B gelatin, and analyzed the effects of the conformational ordering of *κ*-car induced upon cooling in the presence of potassium chloride (KCl) or tetramethylammonium iodide (Me_4_NI). Experimental results showed that the effects of conformational ordering on protein/polyelectrolyte electrostatic complexation can be decomposed into ionic binding and chain stiffening. At the initial stage of conformational ordering, electrostatic complexation can be either suppressed or enhanced due to the ionic bindings of K^+^ and I^−^ ions, which significantly alter the charge density of *κ*-car or occupy the binding sites of gelatin. Beyond a certain stage of conformational ordering, i.e., helix content *θ* > 0.30, the effect of chain stiffening, accompanied with a rapid increase in helix length ζ, becomes dominant and tends to dissociate the electrostatic complexation. The effect of chain stiffening can be theoretically interpreted in terms of double helix association.

Protein/polyelectrolyte electrostatic complexation has been drawing much attention over the past half century. It is essential to many fundamental cellular processes including DNA replication, transcription, and repair^1^,^2^. It has found a wide range of applications in the biotechnological and pharmaceutical industries, such as protein separation and purification, enzyme immobilization^3^, drug delivery and gene therapy^4^. The food industry utilized protein/polyelectrolyte electrostatic complexation for design of low-calorie and low-starch foods^5^, encapsulation of flavors and probiotics^6^,^7^, and stabilization of emulsions and foams^8^.

It is generally envisaged that protein/polyelectrolyte complexation occurs via a two-step process upon changing pH. Intramolecular soluble complexes are first formed, which are followed by the occurrence of intermolecular complexes and insoluble complexes due to increased electrostatic interaction, leading to either a liquid—liquid phase separation or precipitation^9^. Experimental parameters such as pH, ionic strength, ion type, protein/polyelectrolyte ratio, and charge density are well known to influence protein/polyelectrolyte complexation^9^,^10^,^11^. Particularly, the effects of pH and ionic strength have been most extensively studied, as they directly determine the number of charges carried by the protein and polyelectrolyte. In contrast, the effects of conformation and conformational transition have been barely investigated, despite that they are fundamentally important for many natural polyelectrolytes, e.g. the coil-helix transitions of DNA and most polysaccharides^3^,^12^. In principle, conformational transition can alter significantly the chain stiffness and charge density of polyelectrolyte and thus has a great impact on protein/polyelectrolyte electrostatic complexation.

*κ*-Carrageenan (*κ*-car) is a negative polysaccharide extracted from marine red algae, and chemically it is composed of alternating *α*-(1–3)-D-galactose-4-sulfate and *β*-(1–4)-3,6-anhydro-D-galactose repeating units^13^. *κ*-car has been widely used as a thickener, gelling agent, and stabilizer in the food industry^14^. It undergoes a conformational transition from random coil to double helix upon cooling, leading to the formation of an elastic gel^13^. The precise gelation mechanism of κ-car is still controversial, but a two-step mechanism has been generally accepted, which consists of a conformational ordering from coil to double helix and subsequently the aggregaton of the double helices^15^,^16^. The coil-helix-aggregate transition is ion-specific. Certain cations such as K^+^, Cs^+^ and Rb^+^ are known to bind specifically to *κ*-car in the helical state, promoting the double helix formation and aggregation, thus the gelation^17^. On the other hand, some specific anions, notably I^−^, though can bind to and stabilize the double helices, they tend to prevent their aggregation and gelation^15^,^16^. Gelatin is a denatured product from collagen through a hydrolysis process. It exists as a coil at high temperatues and partially renatures into triple helix upon cooling^18^,^19^. Gelatin can be regarded as a special form of protein whose net charge depends on pH^20^.

The objective of the present work is to investigate the dynamic effects of conformational ordering on protein/polyelectrolyte electrostatic complexation, by referring to a mixed system of *κ*-car and type B gelatin. The *κ*-car/gelatin electrostatic complexation was monitored using complementary techniques including turbidimetry, differential scanning calorimetry (DSC), conductivity, fluorescence probing, and confocal laser scanning microscopy (CLSM), during the dynamic process of cooling induced conformational ordering of *κ*-car in the presence of two specific ions, K^+^ and I^−^. The effects of conformational ordering are analyzed in terms of specific ionic binding and chain stiffening both experimentally and theoretically.

## Results

### Electrostatic complexation of *κ*-car/gelatin during cooling in the presence of KCl

**Figure 1:**
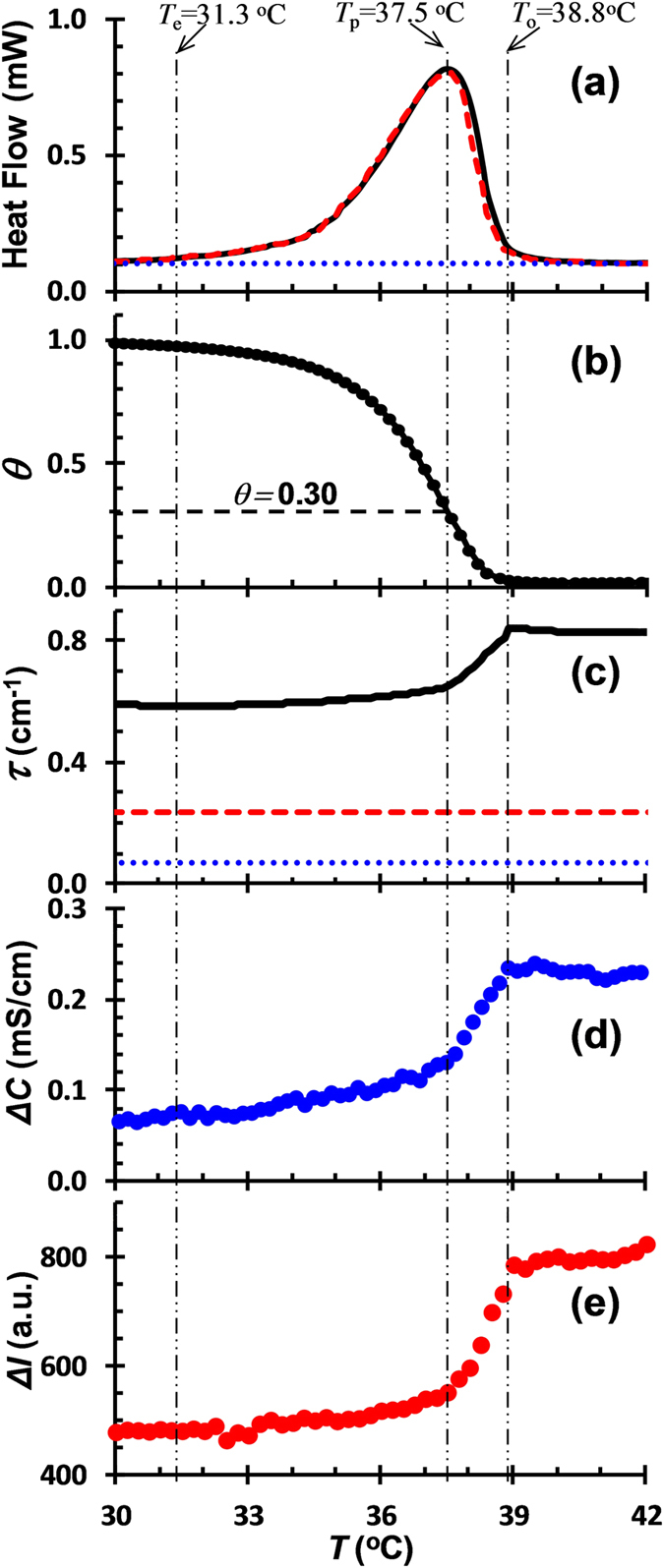
(**a**) DSC curves of 0.75% *κ*-car (dashed line), 0.75% gelatin (dotted line), and 0.75% *κ*-car/0.75% gelatin mixture (solid line) upon cooling; (**b**) *κ*-car helix content *θ* as a function of temperature calculated from DSC according to eq. 2; (**c**) Turbidity *τ* of 0.75% *κ*-car (dashed line), 0.75% gelatin (dotted line), and 0.75% *κ*-car/0.75% gelatin mixture (solid line) upon cooling; (**d**) Difference in conductivity between 0.75% *κ*-car and 0.75% *κ*-car/0.75%gelatin mixture (Δ*C = C*_κ-car_ − *C*_κ-car/gelatin_) as a function of temperature; (**e**) Difference in fluorescence intensity between 0.75% gelatin and 0.75% *κ*-car/0.75% gelatin mixture (Δ*I* = *I*_gelatin_ − *I*_κ-car/gelatin_) as a function of temperature. Cooling rate: 0.5 °C/min; pH: 7.0; Solvent: 50 mM KCl.

Electrostatic complexation of 0.75%*κ*-car/0.75%gelatin mixture during cooling in the presence of KCl, accompanied with the conformational ordering of *κ*-car, was investigated by means of turbidity, DSC, conductivity and fluorescence measurements. The investigations were made in a temperature range above the conformational ordering temperature of gelatin. This avoided the complication by gelatin conformational transition. One typical example at KCl = 50 mM is shown in Fig. 1. The DSC curve of individual gelatin exhibits no thermal transition within the temperature range studied (Fig. 1a), suggesting a coiled conformation^10^. The DSC curve of individual *κ*-car shows a sharp and asymmetric exothermic peak with an onset temperature of *T*_o_ = 38.8 °C, a peak temperature of *T*_p_ = 37.5 °C and an end temperature of *T*_e_ = 31.3 °C. It is ascribed to the coil-to-double helix transition of *κ*-car induced by cooling^17^. The DSC curve of *κ*-car/gelatin mixture is identical to that of pure *κ*-car, indicating that the presence of gelatin has a negligible effect on the conformational ordering of *κ*-car at this specific mixing ratio. The relative values of *κ*-car helix content calculated as a function of temperature, according to eq. (2), are given in Fig. 1b. At *T* = *T*_p_, a helix content of *θ* = 0.30 is formed. This peak temperature corresponds to a maximum in heat flow and thus a maximum in the rate of conformational transition^21^, where helix formation occurs extensively.

Figure 1c shows the turbidity change of individual gelatin and *κ*-car and their mixtures upon cooling in the presence of KCl. Each individual biopolymer solution is almost transparent over the whole temperature range and no considerable change in turbidity is observed upon cooling. The conformational ordering of *κ*-car does not cause any change in turbidity under the experimental conditions. The turbidity of *κ*-car/gelatin mixture is however significantly higher than that of individual biopolymer solution, and it is nearly constant at high temperatures. The appearance of clear turbidity at high temperatures is attributed to the complex coacervation of *κ*-car/gelatin induced by electrostatic interactions^20^,^22^. Although being overall negatively charged, gelatin molecules at pH 7.0 possess local positive patches that could interact with the negatively charged *κ*-car. The association between the positive and negative patches of gelatin molecules seems impossible, since no turbidity was observed in pure gelatin solution at pH 7.0. This might be due to the fact that the electrostatic attraction was not strong enough to override the electrostatic repulsion between negative patches. The electrostatically induced complex coacervation is thought to be less temperature dependent^20^,^23^, and therefore shows a nearly constant turbidity at higher temperatures. With further decreasing the temperature, the turbidity of *κ*-car/gelatin mixture exhibits a complex two-step change accompanying the conformational ordering of *κ*-car. The turbidity first decreases steeply, followed by a second slow decline. The turning point coincides with the peak temperature *T*_p_. The two-step decrease in turbidity suggests that the electrostatic complexation of *κ*-car/gelatin is reduced by two different mechanisms during the conformational ordering of *κ*-car.

Conductivity and fluorescence measurements can be used to probe the complexation of *κ*-car and gelatin. Here, the difference in conductivity between pure *κ*-car solution and *κ*-car/gelatin mixture, Δ*C* = *C*_*κ*-car_−*C*_*κ*-car/gelatin_, is introduced (Fig. 1d). As pure gelatin solution contributes little to the conductivity (<2%), Δ*C* reflects the difference between *κ*-car in free and complex states. As is shown in Fig. 1d, ΔC exhibits a similar two-step change accompanying the conformational ordering of *κ*-car. It first decreases sharply until *T*_p_ and then slowly until *T*_e_. This indicates that the amount of *κ*-car in complex state is reduced in two consecutive steps during the conformational ordering of *κ*-car. Figure 1e plots the difference in fluorescence intensity between pure gelatin solution and *κ*-car/gelatin mixture, Δ*I* = *I*_gelatin_−*I*_κ-car/gelatin_, as a function of temperature. Since only the gelatin is labeled, Δ*I* characterizes the difference between gelatin in free and complex states. Again, a two-step decrease in Δ*I*, marked by *T* = *T*_p_, is observed. This indicates that the amount of gelatin in complex state is reduced accordingly in two steps during the conformational ordering of *κ*-car.

**Figure 2:**
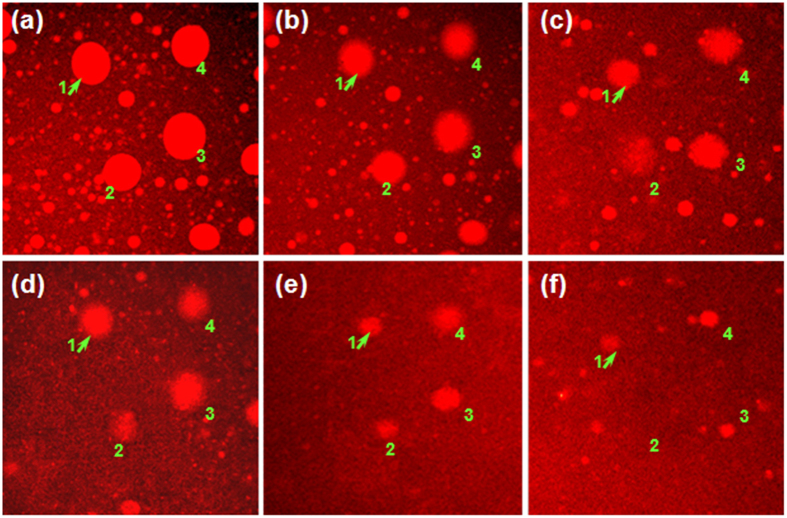
CLSM images of 0.75% *κ*-car/0.75% gelatin mixture in the presence of 50 mM KCl at various temperatures: (**a**) 40.0 °C, (**b**) 38.5 °C, (**c**) 37.5 °C, (**d**) 36.5 °C, (**e**) 35.0 °C and (**f**) 32.0 °C upon cooling. The number “1”, “2”, “3”, and “4” identifies the same object of complex coacervate during the cooling. All the images have a size of 150 × 150 μm.

The turbidity, conductivity and fluorescence measurements jointly reveal that the electrostatic complexation of *κ*-car/gelatin is reduced in two steps during the conformational ordering of *κ*-car in the presence of KCl. This is further supported by CLSM observations on the microstructures of *κ*-car/gelatin complex coacervates during cooling (Fig. 2). The same droplets of complex coacervate show a clear tendency of shrinking with decreasing temperature during the conformational ordering of *κ*-car. *κ*-car/gelatin complex coacervate is clearly dissociated by the conformational ordering of *κ*-car.

### Electrostatic complexation of *κ*-car/gelatin during cooling in the presence of Me_4_NI

**Figure 3:**
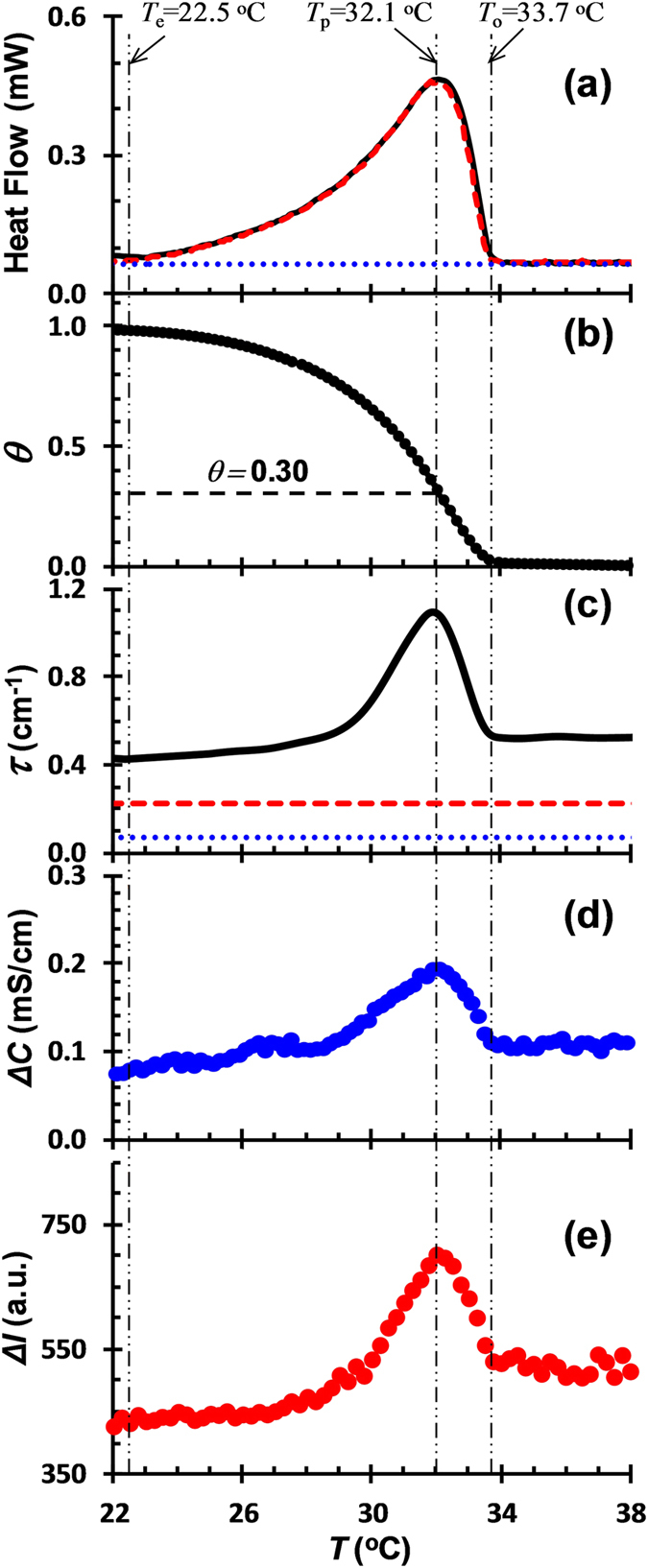
(**a**) DSC curves of 0.75% *κ*-car (dashed line), 0.75% gelatin (dotted line), and 0.75% *κ*-car/0.75% gelatin mixture (solid line) upon cooling; (**b**) *κ*-car helix content *θ* as a function of temperature calculated from DSC according to eq. 2; (**c**) Turbidity *τ* of 0.75% *κ*-car (dashed line), 0.75% gelatin (dotted line), and 0.75% *κ*-car/0.75% gelatin mixture (solid line) upon cooling; (**d**) Difference in conductivity between 0.75% *κ*-car and 0.75% *κ*-car/0.75% gelatin mixture (Δ*C* = *C*_κ-car_ − *C*_κ-car/gelatin_) as a function of temperature; (**e**) Difference in fluorescence intensity between 0.75% gelatin and 0.75% *κ*-car/0.75% gelatin mixture (Δ*I* = *I*_gelatin_ − *I*_κ-car/gelatin_) as a function of temperature. Cooling rate: 0.5 °C/min; pH: 7.0; Solvent: 70 mM Me_4_NI.

The effect of *κ*-car conformational ordering on the electrostatic complexation of *κ*-car/gelatin was investigated in a different salt solution, i.e., Me_4_NI. Figure 3 shows the DSC, turbidity, conductivity and fluorescence results in the presence of 70 mM Me_4_NI upon cooling. At this Me_4_NI concentration, *κ*-car exhibits a sharp and asymmetric exothermic peak during cooling with *T*_o_ = 33.7 °C, *T*_p_ = 32.1 °C and *T*_e_ = 22.5 °C. This transition is attributed to the coil-to-double helix transition of *κ*-car^17^. Pure gelatin solution has no thermal change within the temperature range studied. The calculation of *κ*-car helix content yields *θ* = 0.30 at *T* = *T*_p_ (Fig. 3b). This value is exactly the same with that obtained in KCl solution (Fig. 1b).

Unlike in KCl, the turbidity of *κ*-car/gelatin mixture in Me_4_NI is first increased and then decreased during the conformational ordering of *κ*-car induced by cooling. The turning point is also at *T* = *T*_p_. It is suggested that the electrostatic complexation of *κ*-car/gelatin is first enhanced and then suppressed during the conformational ordering of *κ*-car. The analysis of Δ*C* and Δ*I* leads to the same conclusion (Fig. 3c,d).

**Figure 4:**
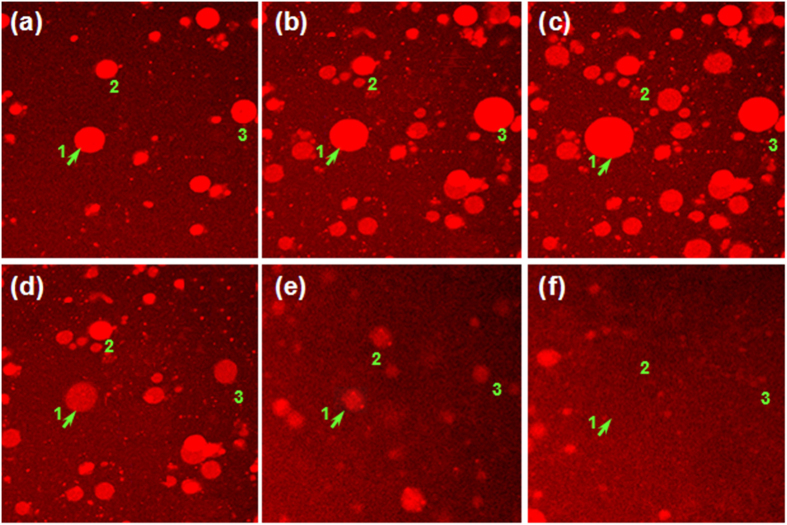
CLSM images of 0.75% *κ*-car/0.75% gelatin mixture in the presence of 70 mM Me_4_NI at various temperatures: (**a**) 35.0 °C, (**b**) 33.0 °C, (**c**) 32.0 °C, (**d**) 31.0 °C, (**e**) 29.0 °C and (**f**) 24.0 °C upon cooling. The number “1”, “2”, and “3” identifies the same object of complex coacervate during the cooling. All the images have a size of 150 × 150 μm.

The microstructures of *κ*-car/gelatin complex coacervates in 70 mM Me_4_NI observed by CLSM at selected temperatures during the conformational ordering of *κ*-car are displayed in Fig. 4. The CLSM images at 35, 33 and 32 °C show a conspicuous growth in coacervate droplets with decreasing temperature (Fig. 4a–c). This is consistent with the first increase in turbidity as observed in Fig. 3c. However, a further decrease in temperature below 32 °C leads to a gradual disappearance of the coacervate droplets (Fig. 4d–f). This corresponds to the turndown of the turbidity in Fig. 3c. Therefore, the conformational ordering of *κ*-car in the presence of Me_4_NI upon cooling first promotes the electrostatic complexation of *κ*-car/gelatin, followed by an opposite effect of dissociation.

### Effect of the concentrations of KCl and Me_4_NI

**Figure 5:**
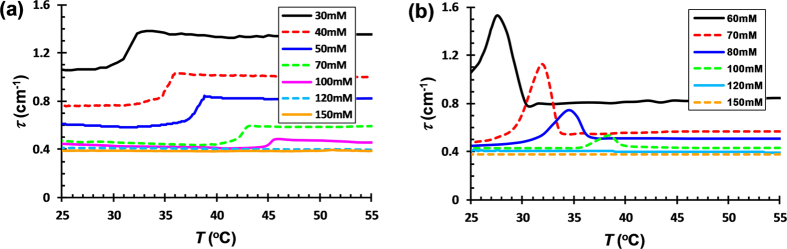
Turbidity change for 0.75% *κ*-car/0.75% gelatin mixtures at various (**a**) KCl and (**b**) Me_4_NI concentrations during cooling. Cooling rate: 0.5 °C/min; pH: 7.0.

Ionic strength has been known to influence the complexation of protein/polyelectrolyte mixture by exerting an electrostatic screening effect^3^. The effect of *κ*-car conformational ordering on the electrostatic complexation of *κ*-car/gelatin was investigated in various KCl and Me_4_NI concentrations. The evolution of turbidity for 0.75% *κ*-car/0.75% gelatin mixture upon cooling with 30–150 mM KCl and 60–150 mM Me_4_NI is shown in Fig. 5a,b. The data at lower KCl (<30 mM) and Me_4_NI (<60 mM) concentrations are not included, as the overlapping of the conformational orderings of *κ*-car and gelatin complicates the situation. Similar to those demonstrated in Figs 1c and 3c, the turbidity of *κ*-car/gelatin mixture displays a two-step decrease in KCl and an increase-decrease change in Me_4_NI upon cooling. Moreover, the turbidity change shifts to higher temperature with increasing KCl and Me_4_NI concentration, and coincides well with the conformational ordering of *κ*-car detected by DSC. It is well known that the conformational transition temperature of *κ*-car increases with the addition of salts such as KCl and Me_4_NI^17^. On the other hand, the extent of turbidity change diminishes with increasing salt concentration, and disappears completely when KCl and Me_4_NI are >100 mM. Meanwhile, the baseline value of the turbidity at higher temperatures decreases with increasing the salt concentrations, and stays at a constant low value when KCl and Me_4_NI are >100 mM. This is due to the electrostatic complex coacervation of *κ*-car/gelatin being reduced with increasing salt concentration and completely screened when KCl and Me_4_NI are >100 mM. Similar effects of ionic strength on *κ*-car/gelatin have been observed in NaCl^10^,^20^. The results at various salt concentrations indicate that the two-step change in turbidity observed during the conformational ordering of *κ*-car in KCl and Me_4_NI is closely associated with the variation in electrostatic complexation of *κ*-car/gelatin. The conformational ordering of *κ*-car itself does not elicit any turbidity change in pure *κ*-car system within the salt concentration range studied.

## Discussion

As demonstrated by DSC, turbidity, conductivity, fluorescence measurements and microstructural observations, the conformational ordering of *κ*-car upon cooling alters significantly the electrostatic complexation of *κ*-car/gelatin system. The effects are found to be dependent on the specific salts used. In the presence of KCl, the conformational ordering of *κ*-car tends to dissociate the electrostatic complexation in a two-step manner. In contrast, the conformation ordering in the presence of Me_4_NI first promotes the electrostatic complexation and then turns to suppress it. Regardless of KCl or Me_4_NI, the two steps are marked by a common boundary, namely, at the helix content *θ* = 0.30.

It is well known that the conformational ordering of *κ*-car upon cooling involves the transition from single coils to double helices and further to a state of aggregated helices^16^,^17^. Electrostatic interaction and hydrogen bonding are believed to play important roles in stabilizing the double helices^17^. Certain cations, such as K^+^, Cs^+^ and Rb^+^, and anions such as I^−^, can bind specifically to *κ*-car helices, and therefore energetically favor and promote their formation^24^. The binding of the different types of ions to *κ*-car helices would certainly alter the charge type and density of *κ*-car. On the other hand, chain stiffness is also believed to change significantly during the coil-to-double helix transition. For example, the persistence length of *κ*-car in coiled and helical conformations was found to be 14 and 60 nm, respectively, in 10 mM NaCl^25^. Depending on NaCl concentration, the chain stiffness increased by 2–3 folds^25^. Therefore, ionic binding and chain stiffening represent the two most important aspects of the conformational ordering of *κ*-car. Their effects on the electrostatic complexation of *κ*-car/gelatin are discussed further in the following sections.

### Ionic binding

Although K^+^ and I^−^ both bind specifically to *κ*-car helices and contribute to their stabilization, they have different interacting modes with *κ*-car helices. In the case of K^+^, the hydrated K^+^ions are small enough to be space-filled between the nearest sulphate groups of two packed *κ*-car chains via electrostatic interaction^26^,^27^,^28^. The three-dimensional ordered packing arrangement allows each sulphate group to be effectively surrounded by K^+^ions and therefore neutralized^26^,^28^. This leads to a reduction in inter-chain electrostatic repulsion and thus the promotion of aggregation of *κ*-car helices^17^. It is anticipated that the ionic binding of K^+^ during the conformational ordering causes the charge annihilation of the negative sulphate groups on the surface of *κ*-car helices. The reduced density of negative charges impairs the electrostatic attraction between *κ*-car and gelatin. This can explain the dissociation of electrostatic complexation of *κ*-car/gelatin at the beginning of the conformational ordering in the presence of KCl and hence the reduced turbidity observed experimentally.

In the case of I^−^, NMR and molecular modeling showed that I^−^ ions can be accommodated into the hydrophobic pockets located at the interior of *κ*-car double helices^29^. The ionic binding of I^−^ ions during the conformation ordering leads to an increased negative charge density of *κ*-car double helices^17^. This might be the reason why I^−^ ions prevent the aggregation of *κ*-car double helices, as the electrostatic repulsion between the double helices would increase accordingly. This can also explain the promotion of electrostatic complexation of *κ*-car/gelatin at the beginning of the conformational ordering in the presence of Me_4_NI, as the electrostatic attraction between *κ*-car and gelatin increases. It is in concert with the increase in turbidity observed experimentally.

In a previous study, we investigated the effect of *κ*-car conformational ordering on the electrostatic complexation of *κ*-car/gelatin in the presence of NaCl^10^,^20^. At the initial stage of the conformational ordering, a decrease in turbidity was also observed (e.g., NaCl = 150 mM), but to a much smaller extent than that found in the presence of KCl. This might be attributed to the non-specific binding of Na^+^ to *κ*-car double helices, which only slightly reduces the charge density of *κ*-car and thus has a limited effect on the dissociation of *κ*-car/gelatin electrostatic complexation. It should be pointed out that K^+^ and I^−^ can also affect the structure of gelatin as a polyelectrolyte. However, in comparison to their specific binding to *κ*-car, the non-specific effect of these ions on gelatin is negligible^18^.

### Chain stiffening

As the conformational ordering of *κ*-car further proceeds, the electrostatic complexation of *κ*-car/gelatin is dissociated by a second step in both KCl and Me_4_NI. The second step starts experimentally at *θ* = 0.30. It is postulated that the dissociation of electrostatic complexation in this step is related to a significant chain stiffening when *κ*-car double helices propagate cooperatively.

**Figure 6:**
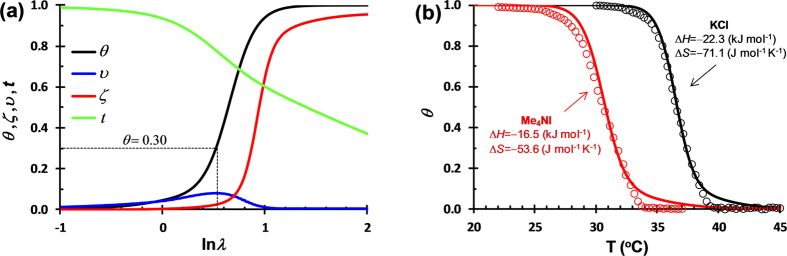
(**a**) Plots of the helix content *θ*, the mean helix length ζ, the number of helical segments on a chain υ, and the probability of a monomer in coiled conformation *t* as a function of the association constant *λ* of *κ*-car double helices. A degree of polymerization of *n* = 572 and a volume fraction of *ϕ* = 0.0113 are used for calculation. ζ and υ have been normalized by *n*. (**b**) Helix content *θ* fitted to the experimental values obtained from DSC (open circles) for *κ*-car in KCl and Me_4_NI, according to the equation ln*λ*(T)=Δ*S*/*R*−Δ*H*/*RT*. Δ*H* and Δ*S* are the enthalpy and entropy for the formation of *κ*-car double helices.

The cooling-induced formation of double helix has been theoretically treated by Tanaka^30^. According to the theory (see supplementary information), the statistic parameters describing the growth of double helices, including the relative helix content *θ*, the normalized mean helix length ζ, the normalized number of helical segments on a chain υ, and the probability of a monomer in coiled conformation *t*, are related to the association constant *λ* of double helices. The *κ*-car sample used in the experiments has a weight-average molecular weight of *M*_w_ = 467 kDa (double strands) and a concentration of 0.75 wt%, corresponding to an average degree of polymerization of *n* = 572 and a *κ*-car volume fraction of *ϕ* = 0.0113. Application of these values yields quantitative relationship between *θ*, ζ, υ, *t* and *λ*, as shown in Fig. 6a. Further considering ln*λ*(*T*) = Δ*S*/*R*−Δ*H*/*RT*, the theoretical values of *θ* can be fitted to those obtained from DSC analysis (Figs 1b and 3b) in KCl and Me_4_NI, respectively, as shown in Fig. 6b.

With decreasing temperature *T*, that is, increasing ln*λ*, the probability of a monomer in coiled conformation *t* decreases, and the relative helix content *θ* increases. The number of helical segments on a chain υ first increases and then decreases. This can be understood as a result of initial formation of interspersing short helical nuclei that beyond a critical size cooperatively propagate into continuous long helices^31^,^32^. This is corroborated by an abrupt increase in helix length ζ at the maximum of υ. Interestingly, the maximum of υ corresponds to a relative helix content of *θ* = 0.30, which agrees exactly with the experimental boundaries observed for the two-step changes in the electrostatic complexation of *κ*-car/gelatin (Figs 1 and 3). The dissociation of electrostatic complexation in the second step therefore should be attributed to a significant chain stiffening that is caused by a sudden increase in helix length during the conformational ordering of *κ*-car. The increase in chain stiffness requires a higher critical surface charge density for the protein and polyelectrolyte to interact electrostatically, and is unfavorable for the electrostatic complexation between *κ*-car/gelatin.

By adjusting Δ*H* and Δ*S*, the enthalpy and entropy of double helix formation, the theoretically calculated *θ* can be fitted reasonably well to those obtained from DSC (Fig. 6b). The small discrepancy might be attributed to the polydispersity of *κ*-car, which is not taken into consideration in the theoretical treatment^30^. The fittings yield Δ*H* = −22.3 kJ mol^−1^ and Δ*S* = −71.1 J mol^−1^ K^−1^ in KCl, and Δ*H* = −16.5 kJ mol^−1^ and Δ*S* = −53.6 J mol^−1^ K^−1^ in Me_4_NI. The enthalpy values are in good agreement with those measured by DSC, i.e., Δ*H* = −20.1 kJ mol^−1^ in KCl and −16.0 kJ mol^−1^ in Me_4_NI. This indicates that the theory proposed by Tanaka is applicable to the double helix formation of *κ*-car induced by cooling. The negative values of Δ*S* are in harmony with the fact that ordered structures are formed during the coil-to-double helix transition of *κ*-car and the freedom of the system is overall reduced.

### Modes of *κ*-car/gelatin electrostatic complexation during conformational ordering and their implications

**Figure 7:**
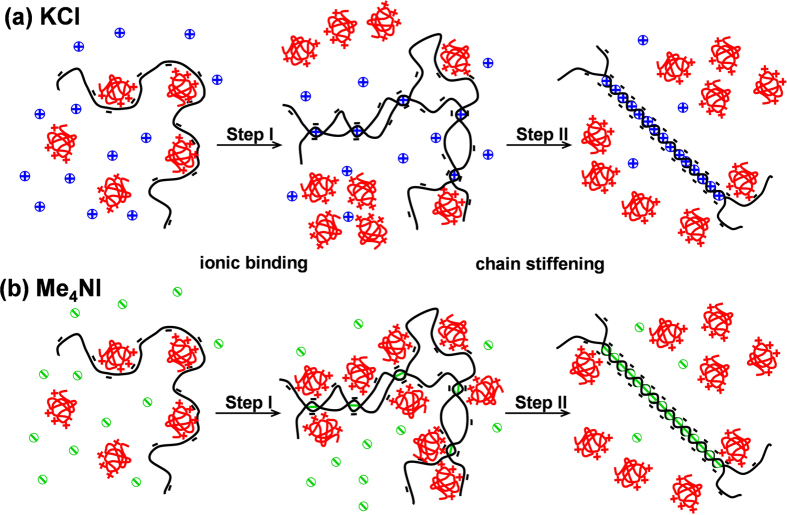
Electrostatic complexation of *κ*-car/gelatin as affected by the conformational ordering of *κ*-car upon cooling in the presence of different salts: (**a**) KCl and (**b**) Me_4_NI. *κ*-car and gelatin molecules are illustrated in black and red, respectively. The blue and green dots represent K^+^ cations and I^−^ anions, repectively.

As discussed above, ionic binding and chain stiffening are the two major processes that exert influences on *κ*-car/gelatin electrostatic complexation during the conformational ordering of *κ*-car. The detailed mechanisms are schematically illustrated in Fig. 7. At the initial stage of conformational ordering (Step I), a limited content of *κ*-car double helices is formed (*θ* < 0.30), resulting in locally ordered helical nuclei. These short helical nuclei do not add much to the chain rigidity and *κ*-car molecules are overall flexible. In this step, specific ionic binding is the dominant effect. Depending on the nature of the specific ions, the binding can either decrease (e.g. K^+^, Fig. 7a) or increase (e.g. I^−^, Fig. 7b) the negative charge density of *κ*-car molecules. Consequently, it leads to a dissociation (Fig. 7a) or promotion (Fig. 7b) of the electrostatic complexation between *κ*-car/gelatin. With a further conversion into double helices, i.e. *θ*  > 0.30 (Step II), the locally ordered helical nuclei cooperatively propagate into continuous long helices. This significantly increases the rigidity of *κ*-car molecules. Chain stiffening comes to dominate in the second step. Although overall being positively charged, gelatin carries both positive and negative charge patches^33^. The rigid structure of *κ*-car allows less freedom in configuration to maximize the electrostatic attraction of *κ*-car/gelatin meanwhile minimizing their electrostatic repulsion^34^. This unfavours the electrostatic complexation of *κ*-car/gelatin and leads to the dissociation of already-formed complexes both in KCl and Me_4_NI (Fig. 7). The inhibitory effect of chain stiffening on electrostatic complexation has been quantitatively described by Mattison *et al*.^35^.

It should be pointed out that the Step I and Step II represent two regimes where specific ionic binding and chain stiffening dominates respectively, and by no means suggests that they are two distinctly separate events. Moreover, the difference between K^+^ and I^−^ ions is that the former promoted the lateral aggregation of *κ*-car helices while the latter inhibited it^17^. The difference in aggregation seems not to produce different impacts on Step II. This suggests that the dissociation of *κ*-car/gelatin complexation in the second step is not determined by the lateral aggregation of *κ*-car double helices.

The effects of ionic binding and chain stiffening on protein/polyelectrolyte electrostatic complexation, as observed during the conformational ordering of polyelectrolyte has important implications in many biological processes and practical applications. In physiological environments, DNA and RNA are constantly surrounded by a layer of mixed ions of various size, charge and specificity (Na^+^, K^+^, Mg^2+^, Ca^2+^, Cl^−^, and different transition metals)^36^,^37^. The so-called “ion atmosphere” is known to dramatically affect the structural stability and functional dynamics of nucleic acids. Particularly, the non-specific binding of cations to the grooves of DNA double helix and the specific binding of some transition metals to defined domains of DNA are essential regulators of DNA-protein interactions and are linked to human disease and health^37^,^38^,^39^.

On the other aspect, DNA structure and topology are temperature-dependent. The decrease in the chain stiffness of DNA at increased physiologically relevant temperatures is important to chromatin organization in *vivo*. It not only leads to a more compact configuration of the genomic DNA but also can have an important effect on cellular processes such as gene regulation and expression^40^. The increase in binding affinity between DNA and architectural proteins, with decreasing chain stiffness, is believed to be the underlying mechanism^40^. Moreover, chain stiffness of polyelectrolyte has been utilized to control the complexation with antibody proteins, and thus to improve the polyelectrolyte-based purification of antibodies^41^.

## Methods

### Materials

Type B bovine bone gelatin (batch no. G9382) was purchased from Sigma Co. Ltd. It has a weight average molar mass of *M*_w_ = 173 kDa, a polydispersity index of *M*_w_/*M*_n_ = 2.0, and a radius of gyration of *R*_g_ = 25.9 nm. The molecular parameters were obtained by gel permeation chromatography coupled with multi-angle laser light scattering (GPC-MALLS) (Wyatt Technology Corporation, USA) in phosphate buffer (1/15 M, pH 7.0) at 25 °C. The isoelectric point (IEP) of the gelatin sample measured by Nano-ZS ZetaSizer (Malvern Instruments, UK) is 5.1, which agrees with the literature values for type B gelatin^20^,^42^. *κ*-car was kindly supplied by FMC biopolymer (Gelcarin GP-911NF). It was converted into sodium type using ion exchange resin (Amberlite IR-120, Sigma), and then lyophilized^10^. The purified sample contains 6.32% Na, 0.067% K, 0.0027% Mg, and 0.0083% Ca, as determined by atomic absorption spectrometry. The molecular parameters measured by GPC-MALLS at 25 °C in 0.1 M NaI are: *M*_w_ = 467 kDa; *M*_w_/*M*_n_ = 1.2; *R*_g_ = 85.0 nm, which represent the double helical conformation of *κ*-car without helical aggregation^16^.

Potassium chloride (KCl) and tetramethylammonium iodide (Me_4_NI) were purchased from Sinopharm Chemical Reagent Co., Ltd. Fluorescein isothiocyanate (FITC) and Rhodamine B were obtained from Sigma-Aldrich, China. All other chemicals, unless otherwise specified, were of analytical grade. Milli-Q water was used in all experiments.

### Sample preparation

Stock solutions of gelatin and *κ*-car at 1.50 wt% were prepared by dissolving appropriate amounts of the samples in KCl or Me_4_NI solutions of various concentrations. The gelatin and *κ*-car dispersions were heated at 60 °C and 85 °C, respectively, for 1 hour under magnetic stirring. The dissolving temperatures were chosen to minimize the possible thermal degradation of gelatin and *κ*-car^20^,^43^. Mixtures of 0.75%*κ*-car/0.75% gelatin were prepared by blending equal amounts of the stock solutions, followed by stirring at 85 °C for 10 mins. The pH of the mixtures were adjusted to pH = 7.0 using 2 M NaOH or HCl. Note that Me_4_NI solutions were always added with 1 mM Na_2_S_2_O_3_ to prevent the oxidation of I^−^ ions^16^.

### Turbidity measurements

Turbidity change at 500 nm as a function of temperature during the cooling of *κ*-car/gelatin solutions was measured on a TU-1900 UV/Vis spectrophotometer (Persee, China). The samples were placed in a copper cuvette fixed with a pair of optical quartz windows. The temperature was controlled by a Peltier device (Quantum Northwest, USA) at a cooling rate of 0.5 °C/min. The turbidity was calculated according to:


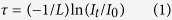


where *L* is the optical path length (1 cm), *I*_0_ the incident light intensity, and *I*_t_ the transmitted light intensity.

### Differential scanning calorimetry (DSC)

DSC measurements were conducted on a high-sensitivity Microcalorimeter DSC-III (Setaram, France). 0.8 g of sample was hermetically sealed into a hastelloy cell and an equal amount of solvent was used as a reference. The sample was heated from room temperature to 70 °C at a scan rate of 3 °C/min and was held at 70 °C for 10 min. The subsequent cooling process from 70 °C to 0 °C at a scan rate of 0.5 °C/min was recorded. The relative value of helix content (*θ*), formed at a certain temperature (*T*), can be calculated from the exothermic DSC peak recorded during cooling^44^,^45^:


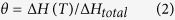


where Δ*H*_total_ and Δ*H*(*T*) represent the total enthalpy change of the conformational ordering of *κ*-car and that up to a temperature *T*. Δ*H*_total_ and Δ*H*(*T*) can be obtained from the integration of the whole DSC peak or partially up to *T*.

### Conductivity measurements

The conductivity of *κ*-car/gelatin solutions was measured using a pH/conductivity meter (Thermo Scientific, USA). The temperature was controlled by a circulating bath AC 200 (Thermo Scientific, USA), and was lowered from 70 °C to 0 °C at a scan rate of 0.5 °C/min.

### Fluorescence measurements

Fluorescence measurements were used to probe the electrostatic complexation of gelatin with *κ*-car. For this purpose, gelatin was labeled with fluorescein isothiocyanate (FITC), according to a method reported previously^46^. In brief, FITC was added to gelatin at a weight ratio of gelatin/FITC = 500 in NaHCO_3_ buffer (pH = 9.0, 0.1 M). The reaction was allowed to proceed overnight at 4 °C in a dark environment with stirring. The solution was dialyzed extensively against PBS and Milli-Q water, followed by filtration (0.45 μm nylon filters) and freeze drying. The fluorescence intensity of 0.75% *κ*-car/0.75% gelatin solutions was measured on a Hitachi F-7000 spectrofluorimeter (Japan). 20% of the total gelatin was replaced with FITC-labeled gelatin. An excitation wavelength of 492 nm and an emission wavelength of 525 nm were used with a bandwidth of 5 nm. The temperature was controlled within ± 0.1 °C by a refrigerated water bath HX-105 (Polycooler, China).

### Confocal laser scanning microscopy (CLSM)

The microstructures of *κ*-car/gelatin solutions upon cooling were observed on a Zeiss LSM 510 META inverted confocal laser scanning microscope (Carl Zeiss AG, Germany), equipped with a multiline argon laser excited at 547 nm. Before blending with *κ*-car, gelatin was stained using 0.02% rhodamine B overnight at ambient temperature. About 5 mL of the mixed solutions was introduced into a home-built temperature-controllable jacketed copper vessel, the bottom of which was made of a thin glass slice (0.5 mm) to facilitate CSLM observations. The temperature was controlled within ± 0.1 °C by a refrigerated water bath HX-105 (Polycooler, China).

## Conclusions

The effects of polyelectrolyte conformational ordering on protein/polyelectrolyte electrostatic complexation have been investigated by using the mixture of *κ*-car/gelatin in KCl and Me_4_NI. Upon the cooling-induced conformational ordering of *κ*-car, the electrostatic complexation of *κ*-car/gelatin undergoes a two-step change, marked by a boundary at *θ* (*κ*-car helix content) = 0.30. Step I sees a dissociation of *κ*-car/gelatin electrostatic complexation in KCl, but a promotion of electrostatic complexation in Me_4_NI. The different effects are due to the specific binding of differently charged K^+^ and I^−^ ions to *κ*-car double helices, leading to an opposite variation in charge density of *κ*-car. Step II experiences a dissociation of *κ*-car/gelatin electrostatic complexation both in KCl and Me_4_NI. Theoretical analysis points to a significant chain stiffening that should be responsible for the dissociation of electrostatic complexation in the step. The detailed modes of *κ*-car/gelatin electrostatic complexation, as influenced by the conformational ordering of *κ*-car, have been proposed. The effects of ionic binding and chain stiffening observed therein have important implications in fundamental biological processes involving DNA-protein interactions and in many practical applications for the pharmaceutical and food industries, etc.

## Additional Information

**How to cite this article**: Cao, Y. *et al*. Effects of conformational ordering on protein/polyelectrolyte electrostatic complexation: ionic binding and chain stiffening. *Sci. Rep.* **6**, 23739; doi: 10.1038/srep23739 (2016).

## Supplementary Material

Supplementary Information
