# Supplementary material for: Effects of conformational ordering on protein/polyelectrolyte electrostatic complexation: ionic binding and chain stiffening
Source: Sci Rep. 2016 Mar 31;6:23739. doi: 10.1038/srep23739 (PMC4814872; doi:10.1038/srep23739)
Supplement: Supplementary Information [file srep23739-s1.doc]

Supplementary Information

Effects of conformational ordering on protein/polyelectrolyte electrostatic complexation: ionic binding and chain stiffening

*Yiping Cao,^1^ Yapeng Fang, ^*, 1,2^ Katsuyoshi Nishinari, ^1,2^ and Glyn O. Phillips ^1^*

^1^ Glyn O. Phillips Hydrocolloid Research Centre, School of Food and Pharmaceutical Engineering, Faculty of Light Industry, Hubei University of Technology, Wuhan 430068, China;

^2^ Hubei Collaborative Innovation Centre for Industrial Fermentation, Hubei University of Technology, Wuhan 430068, China.

^*^To whom correspondence should be addressed: Tel, +86-(0)-27-88015996; Email, [fangypphrc@163.com](mailto:fangypphrc@163.com).

The theory of double helix formation, proposed by Tanaka,^1^ considers that a model polymer chain at a given temperature and concentration first forms partial helical segments after being cooled and then the helical segments from two different chains associate to form double helices (Figure S1).

**Figure S1.** Schematic illustration of double helix formation. Two polymer chains with *n* repeating units associate to form *m* double helices of average length *ζ* . Note that *ζ *is a relative length normalized by *n*.

The association constant *λ*, for the formation of a double helix with mean length *ζ*, is given by

$\ln\lambda\left(T\right)=\Delta S/R-\Delta H/RT$ (1)

where Δ*H* and Δ*S* are the enthalpy and entropy for the association into double helix, and *k_B_* is the Boltzmann constant.

*ζ * is linked to the helix content *θ * and the number of helical segments *υ *on a chain by

$\zeta=\theta/\upsilon$ (2)

*θ* and *υ *are both normalized values by *n*, e.g., *υ *= *m*/*n*, and are given by

$\theta=\left(1-\theta\right)^{2}\varphi\sigma^{2}\lambda t^{4}w_{1}\left(\lambda t^{2}\right)$ (3)

$\upsilon=\left(1-\theta\right)^{2}\varphi\sigma^{2}\lambda t^{4}w_{0}\left(\lambda t^{2}\right)$ (4)

$\left(1-t\right)\left\{1+w_{1}\left(\lambda t^{2}\right)\left(1-t\right)/w_{0}\left(\lambda t^{2}\right)\right\}=\varphi\sigma^{2}\lambda t^{4}w_{0}\left(\lambda t^{2}\right)$ (5)

where *t* is the probability for a randomly chosen monomer to be in coiled conformation, *σ* is the probability for an arbitrarily chosen pair of monomers on two different chains to associate, and *φ *is the polymer volume fraction.

*w_o_* and *w_1_* are defined by

$w_{0}\left(x\right)=\sum_{\zeta=1}^{n} x^{\zeta-1}$ (6)

$w_{1}\left(x\right)=\sum_{\zeta=1}^{n} \zeta x^{\zeta-1}$ (7)

Therefore, *ζ *, *θ*, *υ *and *t* can be calculated from *λ*, which is further linked to temperature *T* according to eq. (1)

**References**

1. Tanaka, F. Thermoreversible gelation driven by coil-to-helix transition of polymers. *Macromolecules* **36**, 5392-5405 (2003).
